# Abstract withdrawn

**DOI:** 10.1186/ar3963

**Published:** 2012-09-27

## Abstract withdrawn

